# Downregulation of nitric oxide by electroacupuncture against hypoxic-ischemic brain damage in rats via nuclear factor-κB/neuronal nitric oxide synthase

**DOI:** 10.3892/mmr.2014.2879

**Published:** 2014-11-06

**Authors:** YICHEN LIU, WEIGUANG LI, LINYAN HU, YING LIU, BAOQUAN LI, CHANGQING SUN, CHENGGANG ZHANG, LIPING ZOU

**Affiliations:** 1 Department of Pediatrics, Chinese PLA General Hospital, Medical School of Chinese PLA, Beijing 100853, P.R. China; 2 Department of Pediatrics, 159th Hospital of Chinese People’s Liberation Army, Zhumadian, Henan 463000, P.R. China; 3 Beijing Institute of Radiation Medicine, State Key Laboratory of Proteomics, Cognitive and Mental Health Research Center of PLA, Beijing 100850, P.R. China

**Keywords:** electroacupuncture, hypoxic-ischemic brain damage, nitric oxide, protection, nuclear factor-κB/neuronal nitric oxide synthase

## Abstract

The present study aimed to investigate the role of nitric oxide (NO) against perinatal hypoxic-ischemic brain damage (HIBD) in rats by electroacupuncture (EA) and to examine its potential neuroprotective mechanism. NO content, the number of positive cells, neuronal nitric oxide synthase (nNOS) and nuclear factor-κB (NF-κB) in rat cortex cells were determined. The results demonstrated that treatment with EA significantly downregulated the NO content in the cortex cells (^*^P<0.05, ^**^P<0.01, compared with the control groups) and alleviated cell damage in the cortex of rats with HIBD. The activator, S-adenosyl-L-methionine and the inhibitor, hydroxylamine of cystathionine-β-synthase (CBS), aggravated and remitted the hypoxic damage in the cortex cells, respectively. In addition, treatment with EA significantly downregulated the expression of nNOS and NF-κB in the rat cortex cells (^*^P<0.05, ^**^P<0.01, compared with the control groups). The results also indicated that treatment with EA downregulated the NO content of cortical cells against HIBD via the NF-κB/nNOS pathway and further implied that the hydrogen sulfide/CBS system may be involved in the process. The present study provided a significant reference for the prevention and treatment of HIBD using the EA technique and also described a novel protective mechanism.

## Introduction

The emerging discipline of gaseous biology in traditional Chinese medicine, has attracted significant attention (1). Various gaseous signaling molecules, including hydrogen sulfide (H_2_S) (2), carbon monoxide (3) and nitric oxide (NO) (4) are involved in regulating homeostasis during acupuncture, in which NO as a messenger has been well-documented under pathological and physiological conditions (5).

NO, also termed endothelium-derived relaxing factor (6), is one of the few gaseous signaling molecules known. NO is biosynthesized from L-arginine, oxygen and nicotinamide adenine dinucleotide phosphate by various nitric oxide synthase (NOS) enzymes *in vivo* (7), including neuronal NO synthase (nNOS), endothelial NO synthase and inducible NO synthase. NO can regulate various biological processes in vertebrates, including the regulation of blood flow (8), blood flow metabolism coupling (9), neurotransmission (10), memory formation (11) and the prevention of apoptosis in neurons (12). In particular, NO is involved in regulating hypoxic-ischemic brain damage (HIBD) (13). Excessive levels of NO can cause reperfusion injury by reacting with superoxide to produce the oxidant peroxynitrite (14), indicating that downregulating the content of NO in cortical cells may facilitate reperfusion injury recovery. Our previous study (15) demonstrated that HIBD upregulates the content of NO in rat cortical cells and that electroacupuncture (EA) can protect this damage by downregulating the NO content in cortical cells. However, the underlying mechanism remains to be elucidated. Therefore, the present study aimed to investigate the potential neuroprotective mechanism of NO downregulation by EA, including the NF-κB/nNOS pathway.

In addition, cystathionine-β-synthase (CBS) is a multi-domain enzyme, located mainly in the brain and nervous system (16,17). It is able to catalyze the transsulfuration pathway to generate H_2_S. H_2_S has various physiological effects, including cysteine S-sulfhydration (18,19), preventing cytokine or oxidant-induced oxidative damage (20), inhibiting the expression of proinflammatory factors by downregulating the activation of NF-κB (21) or upregulating the expression of heme oxygenase 1 (22). Therefore, in the present study it was hypothesized that the NF-κB/nNOS and H_2_S/CBS pathways crosstalk in the HIBD model. Consequently, the CBS activator, S-adenosyl-L-methionine (SAM) and the CBS inhibitor, hydroxylamine (HA) were used on the basis of the HIBD model.

## Materials and methods

### Animals and construction of the HIBD model

A total of 96 specific pathogen-free Sprague-Dawley rats (1 week-old, 12.9–21.0 g) were purchased and raised in the Laboratory Animal Center of the Academy of Military Medical Sciences (Beijing, China). The animals were housed at a temperature of 25±2°C with a 12 h light/dark cycle and were breast fed by their mothers. Each cage contained eight baby rats and their mother. The animals were randomly divided into eight groups (n=12): Sham, Sham + EA, HIBD, HIBD + EA, HIBD + SAM, HIBD + SAM + EA, HIBD + HA and HIBD + HA + EA. The rats were sacrificed using diethyl ether and the four limbs were fixed to enable incision along the neck midline. The left carotid artery communis was exposed through stripping of the thyroid, vein and nervous tissues, which was then ligated using 5/0 surgical line and sutured. After 2 h, the rats were placed in a low-oxygen tank to maintain an appropriate environmental temperature under continuous hypoxia with 8% oxygen and 92% nitrogen for 2 h. The Sham-operated groups were subjected to surgery, which also involved the exposure of the left carotid artery communis, however no ligation was performed. This experiment was approved by the Ethics Committee of the Chinese People’s Liberation Army General Hospital (Beijing, China).

### Intervention experiment of EA

The rats in the EA group were acupunctured at the BaiHui acupoint, which is the crossing point either side of the skull and linkline of the two ears and DaZhui acupoint, which lies below the detail of the cervical spine using EA (~0.25 mm in diameter and 10 mm in length, frequency, 2/100 Hz; intensity, 3 mA) for a 30 min period for 14 days. The limbs of the rats in the control group were simultaneously fixed down, but EA was not performed. The rats in the HIBD + HA and HIBD + HA + EA groups were injected with 12.5 mg/kg/d HA (Sigma-Aldrich, St. Louis, MO, USA), an inhibitor of CBS, via the peritoneal cavity 20 min prior to the acupuncture procedure or fixation. Similarly, the rats in the HIBD + SAM and the HIBD + SAM + EA groups were injected with 50 mg/kg/d SAM (Sigma-Aldrich), an activator of CBS, via the peritoneal cavity 20 min prior to EA or fixation. The control group was injected with an equal volume of normal saline. Subsequently, six rats from each group were sacrificed by cervical dislocation and the brain cortex tissues were obtained to determine the NO content. Tissues from the remaining six animals in each group were perfused with 4% paraformaldehyde for slicing and Nissl and immunohistochemistry (IHC) staining.

### Determination of the NO content in the rat cortex cells

The brain cortex tissue (~50 mg) was homogenize in nine volumes (w/v, ~0.5 ml) of 0.9% ice-cold sodium chloride. The homogenate was centrifuged at 2,500 × g for 10 min and 200 μl supernatant was obtained to measure the protein concentration. The absolute absorbance value (A_550 nm_) was determined according to the manufacturer’s instructions and the NO content was calculated using the following formula: NO = (A_sample_ − A_blank_)/(A_standard_ − A_blank_) × X × Y, where X represents the standard sample concentration (μmol/l) and Y represents the protein concentration (g/l). Column chart analysis was performed using Origin 9.0 software (OriginLab Co., Northampton, MA, USA). Each experiment was repeated at least three times.

### Nissl staining

The slides (3–4 μm) were deparaffinized and rehydrated, following which the frozen or vibratome sections were mounted onto the slides and rehydrated. The sections were partially over-stained with Nissl for ~5 min. The excess stain was removed with tap water, followed by 100% ethanol for 1 min. The sections were transferred to dimethylbenzene (Changhai Chemical Factory, Beijing, China) for 1 h and differentiated with 95% ethanol. The sections were then dehydrated and mounted with neutral balsam. Images of the cortex were captured using a microscope connected to a CCD camera (magnification, ×200; Olympus BX-41; Olypmus, Tokyo, Japan). Each experiment was repeated at least three times.

### IHC assay

The slides (3–4 μm) were deparaffinized, rehydrated, post-fixed with 4% paraformaldehyde for 10 min and then washed three times with 0.01 M phosphate-buffered saline (PBS). Endogenous peroxidase was inactivated by incubating the sections in 3% H_2_O_2_ for 30 min. The sections underwent sequential incubations with 10% normal goat serum in 0.01 M PBS for 30 min at room temperature. The sections were incubated in rabbit anti-nNOS (cat no. ZS-648; 1:100; Beijing Zhongshan, Golden Bridge Biotechnology Co., Ltd, Beijing, China) and rabbit anti-NF-κB (cat no. ab1650; 1:400; Abcam, Cambridge, UK) antibodies in PBS containing 0.3% Triton X-100 overnight at 4°C. Following this, the sections were washed three times with PBS for 5 min each and then incubated in peroxidase-conjugated goat anti-rabbit IgG (1:200; Zymed, San Fransisco, CA, USA) for 1 h at room temperature. Subsequently, the sections were developed with diaminobenzidine (Sigma, St. Louis, MO, USA) in 0.1 M tris-buffered saline containing 0.001% H_2_O_2_ for 30–50 min. Immunoreactions were observed under a microscope (Olympus, Tokyo, Japan). For image analysis, the IHC sections were captured using a microscope connected to a CCD camera (magnification, ×200). Images of five specific areas in each region of the monitor were captured. The quantity of immunopositive cells and total positive area in the assigned subregions was measured using Image-Pro Plus 7.0 software (Media Cybernetics, Inc., Rockville, MD, USA) and column chart analysis was performed using Origin 9.0 software (OriginLab).

### Statistical analysis

All data are expressed as the mean ± standard deviation. Statistical analysis was performed using SPSS software (version 21.0; IBM, Armonk, NY, USA) and Student’s t-test. P<0.05 was considered to indicate a statistically significant difference.

## Results

### Downregulation of NO in the cortex of HIBD rats by EA

**Figure 1:**
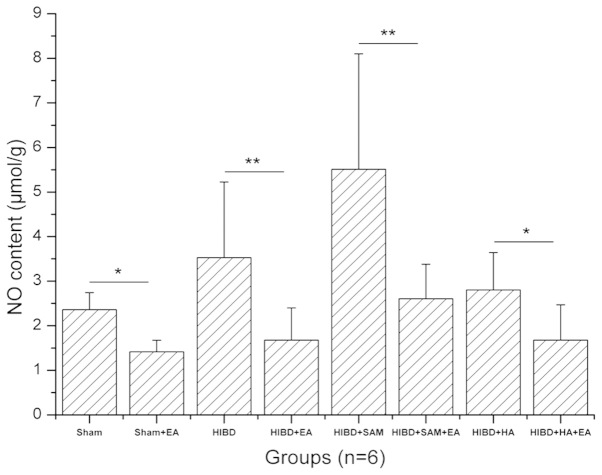
Column chart analysis of the NO content in cortex cells. The chart indicates that EA downregulates the NO content in the cortex cells, with a significant difference compared with each control (^*^P<0.05, ^**^P<0.01). NO, nitric oxide; EA, electroaccupuncture; HIBD, hypoxic-ischemia brain damage; SAM, S-adenosyl-L-methionine; HA, hydroxylamine.

The NO content in the cortex of the Sham, Sham + EA, HIBD, HIBD + EA, HIBD + SAM, HIBD + SAM + EA, HIBD + HA and HIBD + HA + EA groups was 2.3614±0.3807, 1.4165±0.2592, 3.5269±1.6970, 1.6787±0.7213, 5.5101±2.5914, 2.6041±0.7773, 2.8041±0.8377 and 1.6784±0.7917, respectively (Table I). HIBD significantly upregulated the NO content in the cortex cells compared with the Sham group. In addition, treatment with SAM further upregulated the NO content of the cortex cells in the HIBD rats compared with the Sham group. However, treatment with HA downregulated the NO content of the cortex cells compared with the Sham group. Furthermore, EA treatment downregulated the NO content in the Sham + EA, HIBD + EA, HIBD + SAM + EA and HIBD + HA + EA groups compared with those of the control groups, including Sham (^*^P<0.05), HIBD (^**^P<0.01), HIBD + SAM (^**^P<0.01) and HIBD + HA (^*^P<0.05), particularly in the HIBD and HIBD + SAM groups (Fig. 1).

### Alleviation of cell damage in the cortex of the HIBD rats by EA

**Figure 2:**
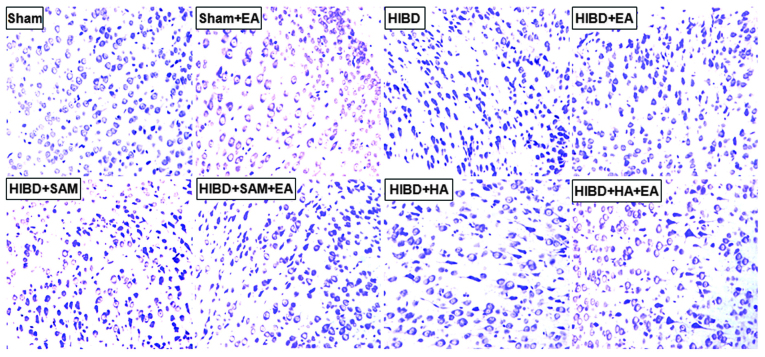
Morphological structure assay of the cortex by Nissl staining. The results indicated that EA is beneficial for alleviating cortex damage in perinatal HIBD rats. EA, electroacupuncture; HIBD, hypoxic-ischemia brain damage; SAM, S-adenosyl-L-methionine; HA, hydroxylamine.

EA treatment alleviated the damage to the cortex in the HIBD rats and decreased the number of positive cells in the Sham + EA, HIBD + EA, HIBD + SAM + EA and HIBD + HA + EA groups compared with those of the control groups, including the Sham, HIBD, HIBD + SAM and HIBD + HA groups (Fig. 2). This result indicated that hypoxia triggered severe damage to the cortex cells, which was aggravated by the CBS activator SAM, but was alleviated by the CBS inhibitor HA (Fig. 2).

### Downregulation of the expression of nNOS in the cortex cells of HIBD rats by EA

**Figure 3:**
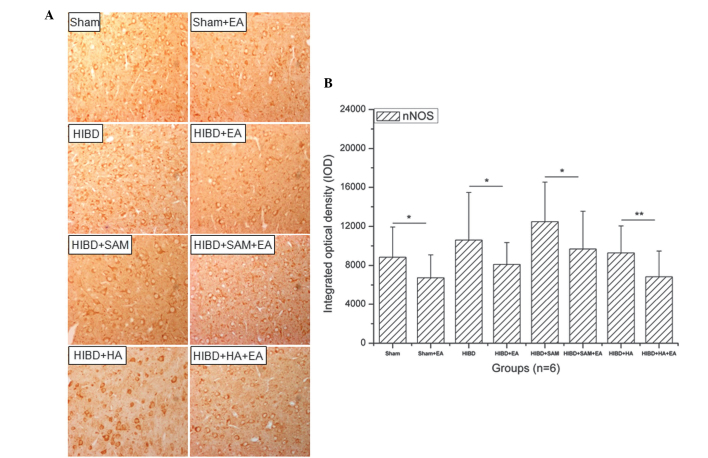
Assay of the level of nNOS expression in the cortex by IHC and column chart analysis. (A) IHC assay of the expression of nNOS in the cortex. (B) Column chart analysis of the expression of nNOS in the cortex. The results indicated that EA downregulates the expression of nNOS in the cortex, with a significant difference compared with each control (^*^P<0.05, ^**^P<0.01). IHC, immunohistochemistry; nNOS, neuronal nitric oxide synthase; EA, electroacupuncture; HIBD, hypoxic-ischemia brain damage; SAM, S-adenosyl-L-methionine; HA, hydroxylamine.

HIBD upregulated the expression of nNOS in the cortex cells compared with the Sham group and treatment with SAM significantly upregulated the expression of nNOS in the cortex cells of the HIBD rats compared with the Sham group. However, treatment with HA downregulated the expression of nNOS in the cortex cells compared with the Sham group. In addition, treatment with EA downregulated the expression of nNOS in the cortex cells of the HIBD rats compared with that of the control groups (Fig. 3A). The expression of nNOS was significantly downregulated following treatment with EA (Fig. 3B). A significant difference in the expression of nNOS was identified between the Sham + EA and the Sham groups (Fig. 3B; ^*^P<0.05). Similarly, a significant difference in the expression of nNOS was also identified between the other EA and the control groups, including HIBD (Fig. 3B; ^*^P<0.05), HIBD + SAM (Fig. 3B; ^*^P<0.05) and HIBD + HA (Fig. 3B; ^*^P<0.01).

### Downregulation in the expression of NF-κB in the cortex cells of HIBD rats by EA

**Figure 4:**
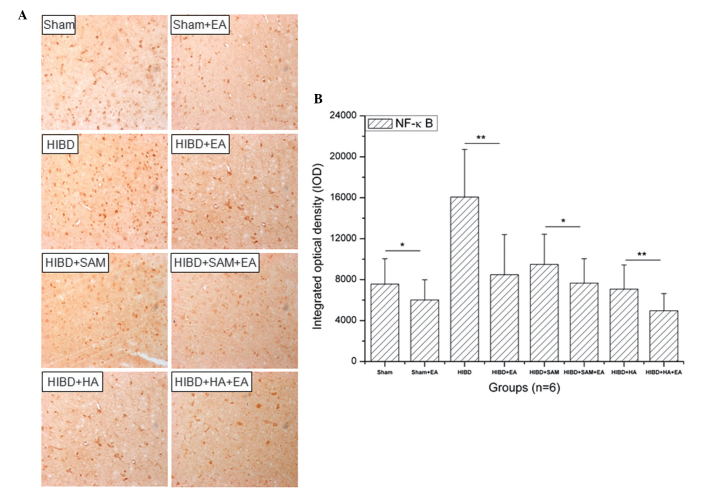
Assay of the expression level of NF-κB in the cortex by IHC and column chart analysis. (A) IHC assay of the expression of NF-κB in the cortex. (B) Column chart analysis of the expression of NF-κB in the cortex. The results indicated that EA downregulates the expression of NF-κB in the cortex, with a significant difference compared with each control (^*^P<0.05, ^**^P<0.01). IHC, immunohistochemistry; NF-κB, nuclear factor-κB; EA, electroacupuncture; HIBD, hypoxic-ischemia brain damage; SAM, S-adenosyl-L-methionine; HA, hydroxylamine.

HIBD upregulated the expression of NF-κB in the cortex cells compared with the Sham group and treatment with SAM significantly upregulated the expression of NF-κB in the cortex cells of HIBD rats compared with Sham treatment. However, treatment with HA downregulated the expression of NF-κB in the cortex cells of HIBD rats compared with the Sham group and treatment with EA downregulated the expression of NF-κB in the cortex cells compared with the control groups (Fig. 4A). A significant difference in the expression of NF-κB was observed between the Sham + EA and the Sham groups (Fig. 4B; ^**^P<0.05). Similarly, a significant difference was identified in the expression of NF-κB between the other EA groups and the control groups, including HIBD (Fig. 4B; ^**^P<0.01), HIBD + SAM (Fig. 4B; ^*^P<0.05) and HIBD + HA (Fig. 4B; ^**^P<0.01).

## Discussion

The present study demonstrated that treatment with EA can downregulate the NO content of cortical cells and alleviate cortex cell damage in HIBD rats. The number of positive cells significantly decreased following treatment with EA compared with each control. In addition, treatment with EA downregulated the expression of nNOS and NF-κB in the rat cortex cells, whereas treatment with SAM significantly upregulated the expression of nNOS and NF-κB in the rat cortex cells. Treatment with HA significantly downregulated the expression of nNOS and NF-κB in the rat cortex cells. These results suggested that the NF-κB/nNOS and H_2_S/CBS pathways may crosstalk during the recovery of HIBD-induced neuron damage.

There are two important mechanisms underlying the regulation of the biological function of NO, namely, S-nitrosation of thiols (23) and nitrosylation of transition metal ions (24). S-nitrosation transfers thiol groups from the cysteine residues of proteins to form S-nitrosothiols and nitrosylation is able to transfer NO to a transition metal ion. Under physiological conditions, NO acts as a tonic inhibitory modulator to regulate carotid body chemosensory discharge by indirectly modifying vascular tone and oxygen delivery and/or directly modulating the excitability of the glomus cells and petrosal neurons (25). In addition, NO has a dual dose-dependent effect on carotid body chemosensory discharge (26).

Acupuncture is used as a curative tool in traditional Chinese medicine and is significantly neuroprotective in organisms through gaseous signaling molecules (27). Acupuncture increases the local generation of NO (4) and increases its content on the surface of the skin at acupoints (28). An increased level of NO has a curative effect on brain damage in rats (29), including transient middle cerebral artery occlusion and HIBD. Acupuncture is also able to cause a decrease in the nNOS/NO system to recover neuronal function, however, the underlying mechanism remains to be elucidated (15). In the present study, treatment with EA downregulated the NO content of the cortical cells in the Sham + EA group as well as following HIBD and treatment with SAM and HA. Treatment with EA also had a significant curative effect on HIBD-induced rat brain damage and downregulated the expression of nNOS and NF-κB in the rat brain. These results suggested that EA may cure brain damage by downregulating the NF-κB/nNOS pathway and that this process is associated with the H_2_S/CBS pathway. However, the present study did not investigate the detailed regulatory association between the NF-κB/nNOS and H_2_S/CBS pathways. Future studies are required to examine this regulatory effect.

In conclusion, the present study revealed that EA can alleviate HIBD in rats by downregulating the NO content of cortex cells. These results provide a significant reference for the prevention and treatment of HIBD using the EA technique and also describe a novel protective mechanism.

## Figures and Tables

**Table I:** NO content assay in rat cortex cells.

Data Groups	NO content (mean ± SD; n=6; μmol/g)
Sham	2.3614±0.3807
Sham + EA	1.4165±0.2592^a^
HIBD	3.5269±1.6970
HIBD + EA	1.6787±0.7213^b^
HIBD + SAM	5.5101±2.5914
HIBD + SAM + EA	2.6041±0.7773^b^
HIBD + HA	2.8041±0.8377
HIBD + HA + EA	1.6784±0.7917^a^

a P<0.05

b P<0.01

SD, standard deviation; NO, nitric oxide; EA, electroaccupuncture; HIBD, hypoxic-ischemia brain damage; SAM, S-adenosyl-L-methionine; HA, hydroxylamine.
